# Meshless Chebyshev RPIM Solution for Free Vibration of Rotating Cross-Ply Laminated Combined Cylindrical-Conical Shells in Thermal Environment

**DOI:** 10.3390/ma15176177

**Published:** 2022-09-05

**Authors:** Zhen Li, Shuangwei Hu, Rui Zhong, Bin Qin, Xing Zhao

**Affiliations:** 1 College of Mechanical and Electrical Engineering, Central South University, Changsha 410083, China; 2 State Key Laboratory of High Performance Complex Manufacturing, Central South University, Changsha 410083, China; 3 Key Laboratory of Traffic Safety on Track, Ministry of Education, School of Traffic & Transportation Engineering, Central South University, Changsha 410075, China; 4 Joint International Research Laboratory of Key Technology for Rail Traffic Safety, Central South University, Changsha 410075, China; 5 National & Local Joint Engineering Research Center of Safety Technology for Rail Vehicle, Central South University, Changsha 410075, China

**Keywords:** meshfree method, laminated composite shell, rotating shell, free vibration analysis, thermal effect

## Abstract

This paper provides a numerical solution to the vibration of a rotating cross-ply laminated combined conical-cylindrical shell in the thermal environment. Its numerical discrete solution method uses the meshless method. The combined shell assumed the temperature independence of material property is divided to the fundamental conical and cylindrical shell substructures, and the theoretical formulation for each substructure is derived based on the first order shear deformation theory (FSDT) and Hamilton’s principle. The effects of the initial hoop tension and temperature change are considered through the kinetic energy reflecting the effects of centrifugal and Coriolis forces and additional strain energy by the nonlinear part of the Green–Lagrange strains. The substructures are then assembled according to the continuity conditions. The boundary and continuity conditions are simulated by introducing artificial virtual spring technology. The displacement component in the theoretical formulation is approximated using a meshless Chebyshev-RPIM shape function. The reliability of the method is verified by comparing with mature and reliable results. The free vibration characteristics of the rotating combined conical-cylindrical shell structure under various sizes, speeds and temperatures are given by numerical examples.

## 1. Introduction

In the aerospace field, laminated shell structures are widely used in the shell structures of gas turbines and high-power aircraft engines [1,2,3,4,5]. In these high-end fields, the vibration of the structure will bring huge economic losses, so it is necessary to study its free vibration behavior before designing such a structure.

With the progress of computational science, many different methods such as the Haar wavelet discretization method [6], geometric analysis (IGA) method [7], spectral-Tchebychev solution technique [8], Ritz method [9,10] and finite element method [11,12,13] are employed for dynamic characteristics analysis of the composite structures. Ye et al. [14] derived the classical open shell formula on the basis of FSDT, and used Chebyshev polynomial to construct the displacement shape function, and solved the free vibration frequency of the open shell through the Rayleigh Ritz program. Caresta and Kessissoglou [15] reported a wave solution for the free vibrational frequencies of a homogeneous composite conical-cylindrical shell, where the displacement component was approximated by a power series. Tornabene et al. [16] reported a method for dynamic analysis of laminated hyperbolic shells and rotating panels on elastic foundations using GDQ. Li et al. [17] reported a Jacobi Ritz method for solving the free vibrations of laminated hyperbolic rotating shells with general boundary constraints. In addition, in recent decades, several studies on the dynamic mechanical properties of rotating structures in thermal environments have been developed. Shakouri et al. [18] reported the vibrational behavior of a conical shell of a functionally graded material with temperature-dependent material properties during rotation. Afshari [19] extended the generalized differential quadrature method to the solution of free vibration of a rotating conical shell reinforced by graphene nanomaterials. Bhangale et al. [20] reported a finite element method for analyzing the dynamics of functionally graded conical shells operating in high temperature environments. Tian et al. [21] obtained the free vibration and forced vibration solutions of the combined conical cylindrical shell by the dynamic stiffness method. Qin et al. [22] used the Rayleigh-Ritz method which based on the energy variation principle to solve the free vibration problem of a cylindrical shell-ring-plate coupling system. Singha et al. [23] analyzed the free vibration characteristics of rotating pretwisted sandwich conical shells in a thermal environment based on high-order shear deformation theory by using a finite element method. Talebitooti et al. [24] investigated the frequency behaviors of the joined conical-conical panel structures based on FSDT by applying Hamilton’s principle. Soureshjani et al. [25] investigated the free vibration behaviors of composite joined conical-conical shell in the thermal environment by using a generalized differential quadrature method. Shi et al. [26] proposed an analytical model for investigating the vibration characteristics of a functionally graded conical-cylindrical coupled shell structure by using a spectro-geometric method. Ghasemi et al. [27] investigated the influences of distribution, mass and volume fractions of fiber, boundary conditions and lay-ups on the sensitivity of vibration behaviors of hybrid laminates cylindrical shell according to Kirchhoff Love’s first approximation shell theory. Liu et al. [28] focused on the influences of rotation on the frequencies and critical speed of CNTs/fiber/polymer/metal laminates cylindrical shell based on Love’s first approximation shell theory. Semnani et al. [29,30] analyzed the vibration behaviors of microshell under varied working conditions by using a finite element method.

In addition to the above methods, the development of meshless theory provides a brand-new idea for plate-shell vibration analysis. Based on the three-dimensional elastic theory, Kwak et al. [31,32] proposed a meshless strong-form solution for the free vibration of laminate shells. In their method, Chebyshev polynomials are introduced as basis functions in the construction of shape functions. In the meshless approach, the establishment of the system algebraic equations of the problem domain does not use a pre-defined mesh for domain discretization, but instead uses nodes [33]. Zarei et al. [34] constructed the displacement function of a prestressed laminate using meshless radial basis point interpolation and analyzed its vibration characteristics. Mellouli et al. [35] used the same method to build a vibrational analysis model of functionally graded carbon nanotube-reinforced shells. Zhang et al. [36] introduced the vibrational behavior of carbon nanotube-enhanced functionally graded triangular plates using a meshless method. Fallah and Delzendeh [37] studied the free vibration of laminates with meshless finite volume method (MFV) as the model solution method and moving least squares approximation to approximate the displacement component. Kwak et al. [38] combined the Chebyshev polynomial with the radial basis point interpolation method to construct the displacement shape function of the open laminated cylindrical shell with elliptical section, and solved its natural frequency.

The purpose of this paper is to study the vibration properties of a rotating cross-laminated conical-cylindrical shell in a thermal environment using meshless theory, considering that the combined structure is divided into cylindrical shell and conical shell structure, and the cylindrical shell is a special conical shell. Therefore, the equations of motion suitable for rotating conical shells are first established within the FSDT framework. Then, the two substructures are assembled by the continuity equation to obtain the equation of the overall structure. The effects of centrifugal force, Coriolis force, and temperature are considered in the equations of motion, and the displacement components involved are approximated using a meshless TRPIM shape function. The accuracy and reliability of the proposed method are verified through the convergence study and comparing with the results of the literature and ABAQUS. Finally, the effects of parameters such as geometry, temperature difference and rotational speed on the free vibration of the cross-ply laminated composite conical-cylindrical shell structure are studied. To sum up, the investigations of this paper can analyze the variation tendency of vibration characteristics of rotating cross-laminated conical-cylindrical shell in the thermal environment and provide the theoretical basis for the designation and manufacture of rotating cross-laminated conical-cylindrical shell structures which are used in aircraft, missiles, submarines, etc.

## 2. Theoretical Formulations

### 2.1. Description of the Model

**Figure 1:**
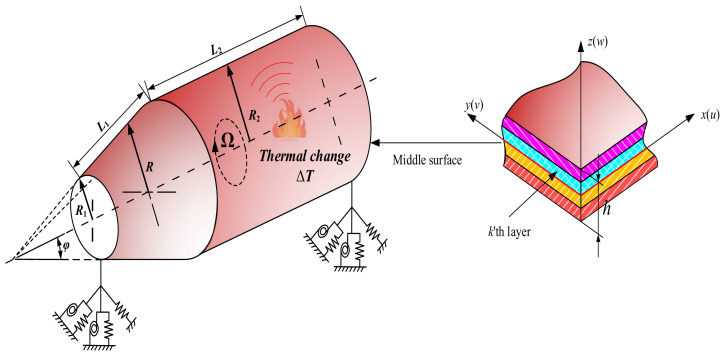
Geometry of rotating cross-ply combined conical-cylindrical shell in thermal environment.

Figure 1 shows a model of laminated combined conical-cylindrical shell rotating with rotating angular velocity Ω under the influence of temperature difference ΔT. The symbols *L*_1_ and *L*_2_ denote the lengths of the two meridians. The thickness of the combined shell is uniformly set to *h*. *φ* represent the semi-vertex angle of conical shell. The symbols *R*_1_ and *R*_2_ represent the radii at both ends of the conical shell, respectively. The cylindrical shell is connected at the big end of the conical shell, so the radius of the cylindrical shell is also *R*_2_. The orthogonal curvilinear coordinate system (*x*, *θ*, *z*) is introduced into the middle surface of each substructure. The orthogonal coordinate system *o*-*xθz* is established on the middle surface of the substructure, then the radius *R* of the random position on the conical shell is as follows:

(1) $$R=R_{1}+x\sin\varphi$$

### 2.2. Governing Equations and Boundary Conditions

According to the assumption of first-order shear deformation [39], the displacement ($\bar{u}$, $\bar{v}$, $\bar{w}$) of any position on the elastic structure can be represented by the displacement (*u*, *v*, *w*, *ψ_x_*, *ψ_θ_*) of the mid-plane.

(2) $$\left\{\begin{array}{c} \bar{u}(x,\theta ,z,t)=u(x,\theta ,t)+z\psi_{x}(x,\theta ,t)\\ \bar{v}(x,\theta ,z,t)=v(x,\theta ,t)+z\psi_{\theta }(x,\theta ,t)\\ \bar{w}(x,\theta ,z,t)=w(x,\theta ,t)\;\;\;\;\; \end{array}\right.$$

Combined with the linear elasticity theory, the relationship between the stress and displacement of the shell is defined:

(3) $$\begin{array}{cc} \; & \left\{\begin{array}{c} \varepsilon_{\alpha }^{0}\\ \varepsilon_{\beta }^{0}\\ \gamma_{\alpha \beta }^{0} \end{array}\right\}=\left\{\begin{array}{c} \frac{1}{A}\frac{\partial u}{\partial \alpha }+\frac{v}{AB}\frac{\partial A}{\partial \beta }+\frac{w}{R_{\alpha }}\\ \frac{1}{B}\frac{\partial v}{\partial \beta }+\frac{u}{AB}\frac{\partial B}{\partial \alpha }+\frac{w}{R_{\beta }}\\ \frac{1}{A}\frac{\partial v}{\partial \alpha }-\frac{u}{AB}\frac{\partial A}{\partial \beta }+\frac{1}{B}\frac{\partial u}{\partial \beta }-\frac{v}{AB}\frac{\partial B}{\partial \alpha } \end{array}\right\},\\ \; & \left\{\begin{array}{c} \chi_{\alpha }\\ \chi_{\beta }\\ \chi_{\alpha \beta } \end{array}\right\}=\left\{\begin{array}{c} \frac{1}{A}\frac{\partial \psi_{\alpha }}{\partial \alpha }+\frac{\psi_{\beta }}{AB}\frac{\partial A}{\partial \beta }\\ \frac{1}{B}\frac{\partial \psi_{\beta }}{\partial \beta }+\frac{\psi_{\alpha }}{AB}\frac{\partial B}{\partial \alpha }\\ \frac{1}{A}\frac{\partial \psi_{\beta }}{\partial \alpha }-\frac{\psi_{\alpha }}{AB}\frac{\partial A}{\partial \beta }+\frac{1}{B}\frac{\partial \psi_{\alpha }}{\partial \beta }-\frac{\psi_{\beta }}{AB}\frac{\partial B}{\partial \alpha } \end{array}\right\},\;\;\left\{\begin{array}{c} \gamma_{\beta z}^{0}\\ \gamma_{\alpha z}^{0} \end{array}\right\}=\left\{\begin{array}{c} \frac{1}{B}\frac{\partial w}{\partial \beta }+\psi_{\beta }\\ \frac{1}{A}\frac{\partial w}{\partial \alpha }+\psi_{\alpha } \end{array}\right\} \end{array}$$

where $\varepsilon ={\left\{\varepsilon_{\alpha }^{0},\varepsilon_{\beta }^{0},\gamma_{\alpha \beta }^{0}\right\}}^{T}$ represents the normal strain and shear strain of the elastic element, and $\chi ={\left\{\chi_{\alpha },\chi_{\beta },\chi_{\alpha \beta }\right\}}^{T}$ represents the bending and torsional curvature changes of the elastic body. $\gamma ={\left\{\gamma_{\beta z}^{0},\gamma_{\alpha z}^{0}\right\}}^{T}$ denotes transverse shear strain. *A* and *B* denote the Lamé parameters.

(4) $$\begin{array}{c} \begin{array}{cc} \; & \mathrm{conical}\;\mathrm{shell}:\alpha =x,\;\beta =\theta ,\;A=1,\;B=R,\;R_{\alpha }=\infty ,\;R_{\beta }=R/\cos\varphi \;\\ \; & \mathrm{cylindrical}\;\mathrm{shell}:\alpha =x,\;\beta =\theta ,\;A=1,\;B=R,\;R_{\alpha }=\infty ,\;R_{\beta }=R \end{array} \end{array}$$

The matrix form of the stress resultants-strain relationship of moderately thick cross-ply conical shell is as

(5) $$\left[\begin{array}{c} N\\ M\\ Q \end{array}\right]=\left[\begin{array}{ccc} A & B & 0\\ B & D & 0\\ 0 & 0 & A_{c} \end{array}\right]\left[\begin{array}{c} \varepsilon \\ \chi \\ \gamma \end{array}\right]$$

where $N={\left\{N_{\alpha },N_{\beta },N_{\alpha \beta }\right\}}^{T}$, the internal element represents the in-plane force. $M={\left\{M_{\alpha },M_{\beta },M_{\alpha \beta }\right\}}^{T}$, the element represents the bending moment, and $Q={\left\{Q_{\beta },Q_{\alpha }\right\}}^{T}$ is the shear force vector. ***A*** represents the tensile stiffness matrix, ***B*** is the bending stiffness matrix, and ***D*** is the coupled tensile bending stiffness matrix. ***A_c_*** denotes the shear stiffness matrix. Their specific form is:

(6) $$\begin{array}{c} \begin{array}{cc} \; & A=\sum_{k=1}^{N}\left[\begin{array}{ccc} {\bar{Q}}_{11}^{k} & {\bar{Q}}_{12}^{k} & {\bar{Q}}_{16}^{k}\\ {\bar{Q}}_{12}^{k} & {\bar{Q}}_{22}^{k} & {\bar{Q}}_{26}^{k}\\ {\bar{Q}}_{16}^{k} & {\bar{Q}}_{26}^{k} & {\bar{Q}}_{66}^{k} \end{array}\right]\left(z_{k+1}-z_{k}\right),B=\frac{1}{2}\sum_{k=1}^{N}\left[\begin{array}{ccc} {\bar{Q}}_{11}^{k} & {\bar{Q}}_{12}^{k} & {\bar{Q}}_{16}^{k}\\ {\bar{Q}}_{12}^{k} & {\bar{Q}}_{22}^{k} & {\bar{Q}}_{26}^{k}\\ {\bar{Q}}_{16}^{k} & {\bar{Q}}_{26}^{k} & {\bar{Q}}_{66}^{k} \end{array}\right]\left(z_{k+1}^{2}-z_{k}^{2}\right)\\ \; & D=\frac{1}{3}\sum_{k=1}^{N}\left[\begin{array}{ccc} {\bar{Q}}_{11}^{k} & {\bar{Q}}_{12}^{k} & {\bar{Q}}_{16}^{k}\\ {\bar{Q}}_{12}^{k} & {\bar{Q}}_{22}^{k} & {\bar{Q}}_{26}^{k}\\ {\bar{Q}}_{16}^{k} & {\bar{Q}}_{26}^{k} & {\bar{Q}}_{66}^{k} \end{array}\right]\left(z_{k+1}^{3}-z_{k}^{3}\right),A_{c}=k_{c}\sum_{k=1}^{N}\left[\begin{array}{cc} {\bar{Q}}_{44}^{k} & {\bar{Q}}_{45}^{k}\\ {\bar{Q}}_{45}^{k} & {\bar{Q}}_{55}^{k} \end{array}\right]\left(z_{k+1}-z_{k}\right) \end{array} \end{array}$$

$$\begin{array}{l} Q_{11}^{k}=\frac{E_{1}^{k}}{1-\mu_{12}^{k}\mu_{21}^{k}},\;Q_{12}^{k}=\mu_{21}^{k}Q_{11}^{k},\;Q_{22}^{k}=\frac{E_{2}^{k}}{1-\mu_{12}^{k}\mu_{21}^{k}},\;Q_{44}^{k}=G_{23}^{k}\\ Q_{55}^{k}=G_{13}^{k},\;Q_{66}^{k}=G_{12}^{k} \end{array}$$

where *N* denotes the number of laying layers of the laminate, *k_c_* = 5/6 is the shear correction coefficient and the symbol ${\bar{Q}}_{ij}^{k}$ denotes the elastic stiffness coefficient [38].

In the thermal environment, the thermal stress of the *k*th layer in the cross-layer is expressed as follows:

(7) $$\left\{\begin{array}{c} \sigma_{x}^{Tk}\\ \sigma_{\theta }^{Tk}\\ \tau_{x\theta }^{Tk} \end{array}\right\}=-\left[\begin{array}{ccc} Q_{11}^{k} & Q_{12}^{k} & 0\\ Q_{12}^{k} & Q_{22}^{k} & 0\\ 0 & 0 & Q_{66}^{k} \end{array}\right]\left\{\begin{array}{c} \alpha_{11}^{k}\Delta T\\ \alpha_{22}^{k}\Delta T\\ \alpha_{12}^{k}\Delta T \end{array}\right\}\;\left[\begin{array}{l} Q_{cxx}^{}\\ Q_{c\theta \theta }^{} \end{array}\right]=\kappa_{s}\left[\begin{array}{cc} A_{44} & A_{45}\\ A_{45} & A_{55} \end{array}\right]\left\{\begin{array}{c} \begin{array}{c} \gamma_{\theta r0}^{p}\\ \gamma_{xr0}^{p} \end{array} \end{array}\right\}$$

where the thermal expansion coefficients $\alpha_{ij}^{k}$ of the *k*th layer are given by

(8) $$\left\{A_{ij},B_{ij},D_{ij}\right\}=\sum_{k=1}^{N_{k}}\int_{z_{k}}^{z_{k+1}}{\bar{Q}}_{ij}^{k}\left\{1,z,z^{2}\right\}dz$$

where *δ_k_* and *α_ij_* denote the fiber angle of the *k*th layer and linear thermal expansion coefficients along the principal axes of a layer, respectively.

The thermal strain can be written as nonlinear part of Green–Lagrange strain.

(9) $$\begin{array}{c} \begin{array}{cc} \; & \varepsilon_{\alpha }^{NL}={\left(\frac{1}{A}\frac{\partial \bar{u}}{\partial \alpha }+\frac{1}{AB}\frac{\partial A}{\partial \beta }\bar{v}+\frac{\bar{w}}{R_{\alpha }}\right)}^{2}+{\left(\frac{1}{A}\frac{\partial \bar{v}}{\partial \alpha }-\frac{1}{AB}\frac{\partial A}{\partial \beta }\bar{u}\right)}^{2}+{\left(\frac{1}{A}\frac{\partial \bar{w}}{\partial \alpha }-\frac{\bar{u}}{R_{\alpha }}\right)}^{2}\\ \; & \varepsilon_{\beta }^{NL}={\left(\frac{1}{B}\frac{\partial \bar{u}}{\partial \beta }-\frac{1}{AB}\frac{\partial B}{\partial \alpha }\bar{v}\right)}^{2}+{\left(\frac{1}{B}\frac{\partial \bar{v}}{\partial \beta }+\frac{1}{AB}\frac{\partial B}{\partial \alpha }\bar{u}+\frac{\bar{w}}{R_{\beta }}\right)}^{2}+{\left(\frac{1}{B}\frac{\partial \bar{w}}{\partial \beta }-\frac{\bar{v}}{R_{\beta }}\right)}^{2}\\ \; & \gamma_{\alpha \beta }^{NL}=\left(\frac{1}{A}\frac{\partial \bar{u}}{\partial \alpha }+\frac{1}{AB}\frac{\partial A}{\partial \beta }\bar{v}+\frac{\bar{w}}{R_{\alpha }}\right)\left(\frac{1}{B}\frac{\partial \bar{u}}{\partial \beta }-\frac{1}{AB}\frac{\partial B}{\partial \alpha }\bar{v}\right)+\\ \; & \;\;\left(\frac{1}{A}\frac{\partial \bar{v}}{\partial \alpha }-\frac{1}{AB}\frac{\partial A}{\partial \beta }\bar{u}\right)\left(\frac{1}{B}\frac{\partial \bar{v}}{\partial \beta }+\frac{1}{AB}\frac{\partial B}{\partial \alpha }\bar{u}+\frac{\bar{w}}{R_{\beta }}\right)+\left(\frac{1}{A}\frac{\partial \bar{w}}{\partial \alpha }-\frac{\bar{u}}{R_{\alpha }}\right)\left(\frac{1}{B}\frac{\partial \bar{w}}{\partial \beta }-\frac{\bar{v}}{R_{\beta }}\right) \end{array} \end{array}$$

Substituting Equations (2) and (14) into Equation (13)

(10) $$\varepsilon_{x}^{NL}={\left(\frac{\partial u}{\partial x}\right)}^{2}+{\left(\frac{\partial v}{\partial x}\right)}^{2}+{\left(\frac{\partial w}{\partial x}\right)}^{2}+z^{2}{\left(\frac{\partial \psi_{x}}{\partial x}\right)}^{2}+z^{2}{\left(\frac{\partial \psi_{\theta }}{\partial x}\right)}^{2}+2z\frac{\partial u}{\partial x}\frac{\partial \psi_{x}}{\partial x}+2z\frac{\partial v}{\partial x}\frac{\partial \psi_{\theta }}{\partial x}$$

$$\begin{array}{c} \begin{array}{cc} \; & \varepsilon_{\theta }^{NL}=\frac{1}{R^{2}}\left[{\left(\frac{\partial u}{\partial \theta }\right)}^{2}+{\left(\frac{\partial v}{\partial \theta }\right)}^{2}+{\left(\frac{\partial w}{\partial \theta }\right)}^{2}+2\sin\varphi \left(\frac{u\partial v}{\partial \theta }-\frac{v\partial u}{\partial \theta }\right)+2\cos\varphi \left(\frac{w\partial v}{\partial \theta }-\frac{v\partial w}{\partial \theta }\right)\right.\\ \; & +u^{2}\sin^{2}\varphi +v^{2}+w^{2}\cos^{2}\varphi +2uw\sin\varphi \cos\varphi +2z\left(\frac{\partial u}{\partial \theta }\frac{\partial \psi_{x}}{\partial \theta }+\frac{\partial v}{\partial \theta }\frac{\partial \psi_{\theta }}{\partial \theta }\right)\\ \; & +2z\sin\varphi \left(\frac{u\partial \psi_{\theta }}{\partial \theta }-\frac{\psi_{\theta }\partial u}{\partial \theta }+\frac{\psi_{x}\partial v}{\partial \theta }-\frac{v\partial \psi_{x}}{\partial \theta }\right)+2z\cos\varphi \left(\frac{w\partial \psi_{\theta }}{\partial \theta }-\frac{\psi_{\theta }\partial w}{\partial \theta }\right)+2zu\psi_{x}\sin^{2}\varphi \\ \; & +2zv\psi_{\theta }+2zw\psi_{x}\sin\varphi \cos\varphi +z^{2}{\left(\frac{\partial \psi_{x}}{\partial \theta }\right)}^{2}+z^{2}{\left(\frac{\partial \psi_{\theta }}{\partial \theta }\right)}^{2}+2z^{2}\sin\varphi \left(\frac{\psi_{x}\partial \psi_{\theta }}{\partial \theta }-\frac{\psi_{\theta }\partial \psi_{x}}{\partial \theta }\right)\\ \; & \left.+z^{2}\psi_{x}^{2}\sin^{2}\varphi +z^{2}\psi_{\theta }^{2}\right] \end{array} \end{array}$$

$$\begin{array}{c} \begin{array}{cc} \; & \gamma_{x\theta }^{NL}=\frac{1}{R}\left[\frac{\partial u}{\partial x}\frac{\partial u}{\partial \theta }\right.+\frac{\partial v}{\partial x}\frac{\partial v}{\partial \theta }+\frac{\partial w}{\partial x}\frac{\partial w}{\partial \theta }+z\frac{\partial u}{\partial x}\frac{\partial \psi_{x}}{\partial \theta }+z\frac{\partial u}{\partial \theta }\frac{\partial \psi_{x}}{\partial x}+z\frac{\partial v}{\partial x}\frac{\partial \psi_{\theta }}{\partial \theta }+z\frac{\partial v}{\partial \theta }\frac{\partial \psi_{\theta }}{\partial x}\\ \; & +z^{2}\frac{\partial \psi_{x}}{\partial x}\frac{\partial \psi_{x}}{\partial \theta }+z^{2}\frac{\partial \psi_{\theta }}{\partial x}\frac{\partial \psi_{\theta }}{\partial \theta }+\sin\varphi \frac{u\partial v}{\partial x}-\sin\varphi \frac{v\partial u}{\partial x}-\cos\varphi \frac{v\partial w}{\partial x}+\cos\varphi \frac{w\partial v}{\partial x}\\ \; & +z\sin\varphi \frac{u\partial \psi_{\theta }}{\partial x}-z\sin\varphi \frac{\psi_{\theta }\partial u}{\partial x}+z\sin\varphi \frac{\psi_{x}\partial v}{\partial x}-z\sin\varphi \frac{v\partial \psi_{x}}{\partial x}+z\cos\varphi \frac{w\partial \psi_{\theta }}{\partial x}\\ \; & -z\cos\varphi \frac{\psi_{\theta }\partial w}{\partial x}\left.+z^{2}\sin\varphi \frac{\psi_{x}\partial \psi_{\theta }}{\partial x}-z^{2}\sin\varphi \frac{\psi_{\theta }\partial \psi_{x}}{\partial x}\right] \end{array} \end{array}$$

In the thermal field, the strain energy of the structure is expressed as:

(11) $$\begin{array}{l} U=U_{e}+U_{T}=\frac{1}{2}\iint_{\Omega }\left(\begin{array}{l} N_{x}\varepsilon_{x}^{0}+N_{\theta }\varepsilon_{\theta }^{0}+N_{x\theta }\gamma_{x\theta }^{0}+M_{x}\chi_{x}\\ +M_{\theta }\chi_{\theta }+M_{x\theta }\chi_{x\theta }+Q_{\theta }\gamma_{\theta z}+Q_{x}\gamma_{xz} \end{array}\right)Rdxd\theta \\ +\frac{1}{2}\iint_{\Omega }\sum_{k=1}^{N_{k}}\int_{Z_{k}}^{Z_{k+1}}\left(\sigma_{x}^{Tk}\varepsilon_{x}^{NL}+\sigma_{\theta }^{Tk}\varepsilon_{\theta }^{NL}+2\tau_{x\theta }^{Tk}\gamma_{x\theta }^{NL}\right)Rdzdxd\theta \end{array}$$

Meanwhile, when the shell rotates, an initial hoop tension will be generated due to centrifugal force, which will generate a part of the strain energy.

(12) $$U_{h}=\frac{1}{2}\int_{0}^{2\pi }\int_{0}^{L}N_{\theta }^{0}\varepsilon_{\theta }^{NL}Rdxd\theta$$

where $N_{\theta }^{0}=\rho h\Omega^{2}R^{2}$ is the initial hoop tension, the unit of *ρ* is kg/m^3^.

The kinetic energy is

(13) $$T=\frac{1}{2}\int_{0}^{h}\int_{0}^{2\pi }\int_{0}^{L}\rho \vec{v}\cdot \vec{v}Rdxd\theta dz$$

where $\vec{v}$ is absolute velocity vector.

(14) $$\vec{v}=\dot{\vec{r}}+\Omega (-\cos\varphi \vec{i}+\sin\varphi \vec{k})\times \vec{r}$$

where $\vec{r}=\bar{U}\vec{i}+\bar{V}\vec{j}+\bar{W}\vec{k}$ is the displacement vector.

When the shell is not affected by external force, according to the variational principle, the equilibrium equation and boundary conditions of the heated rotating cross-layer shell are deduced.

(15) $$\delta \int_{t_{1}}^{t_{2}}\left(T-U-U_{h}\right)dt=0$$

The obtained governing equations are expressed as:

(16) $$Ku+C\dot{u}+M\ddot{u}=0$$

where the matrices ***C*** and ***M*** are expressed as:

(17) $$M=\left[\begin{array}{ccccc} -I_{0} & 0 & 0 & -I_{1} & 0\\ 0 & -I_{0} & 0 & 0 & -I_{1}\\ 0 & 0 & -I_{0} & 0 & 0\\ -I_{1} & 0 & 0 & -I_{2} & 0\\ 0 & -I_{1} & 0 & 0 & -I_{2} \end{array}\right]\;C=\left[\begin{array}{ccccc} 0 & 2I_{0}\Omega \sin\varphi & 0 & 0 & 2I_{1}\Omega \sin\varphi \\ -2I_{0}\Omega \sin\varphi & 0 & -2I_{0}\Omega \cos\varphi & -2I_{1}\Omega \sin\varphi & 0\\ 0 & 2I_{0}\Omega \cos\varphi & 0 & 0 & 2I_{1}\Omega \cos\varphi \\ 0 & 2I_{1}\Omega \sin\varphi & 0 & 0 & 2I_{2}\Omega \sin\varphi \\ -2I_{1}\Omega \sin\varphi & 0 & -2I_{1}\Omega \cos\varphi & -2I_{2}\Omega \sin\varphi & 0 \end{array}\right]$$

where the inertia terms are

(18) $$[I_{0},I_{1},I_{2}]=\int_{-h/2}^{h/2}\rho [1,z,z^{2}]dz$$

The boundary conditions obtained from Hamilton’s principle are expressed as:

(19) $$B_{c}u=0$$

Combining Equations (21), (24) and (25), we can derive the governing equations and boundary conditions for the cross-layer shell in the thermal physics field.

(20) $$\left\{\begin{array}{l} u\;(x,\theta ,t)=U(x)\cos(n\theta +\omega t)\\ v\;(x,\theta ,t)=V(x)\sin(n\theta +\omega t)\\ w\;(x,\theta ,t)=W(x)\cos(n\theta +\omega t)\\ \psi_{x}\;(x,\theta ,t)=\Psi_{x}(x)\cos(n\theta +\omega t)\\ \psi_{\theta }\;(x,\theta ,t)=\Psi_{\theta }(x)\sin(n\theta +\omega t) \end{array}\right.$$

where *ω* and *n* denote the natural frequency and circumferential wave number, respectively.

Substituting Equation (20) into Equations (21) and (24), the one-dimensional governing equations and boundary conditions of rotating cross-ply conical shell in thermal environment are obtained.

(21) $$\left(K_{x}+\omega C_{x}-\omega^{2}m\right)U=0$$

(22) $$B_{x}U=0$$

where

(23) $$U={\left[U(x)\;V(x)\;W(x)\;\Psi_{x}(x)\;\Psi_{\theta }(x)\right]}^{T}$$

(24) $$C_{x}=\left[\begin{array}{ccccc} 0 & -2I_{0}\Omega \sin\varphi & 0 & 0 & -2I_{1}\Omega \sin\varphi \\ -2I_{0}\Omega \sin\varphi & 0 & -2I_{0}\Omega \cos\varphi & -2I_{1}\Omega \sin\varphi & 0\\ 0 & -2I_{0}\Omega \cos\varphi & 0 & 0 & -2I_{1}\Omega \cos\varphi \\ 0 & -2I_{1}\Omega \sin\varphi & 0 & 0 & -2I_{2}\Omega \sin\varphi \\ -2I_{1}\Omega \sin\varphi & 0 & -2I_{1}\Omega \cos\varphi & -2I_{2}\Omega \sin\varphi & 0 \end{array}\right]$$

### 2.3. Meshfree TRPIM Shape Function

The radial point interpolation method is a newly developed meshless method, which is an important and widely used method for solving partial differential equations. The unknown displacement function *u*(*x*) is approximated by using the RPIM difference of the polynomials and can be defined as in [38].

(25) $$u(x)=\sum_{i=1}^{n_{r}}R_{i}(x)a_{i}+\sum_{j=1}^{n_{p}}p_{j}(x)b_{j}=R^{T}(x)a+P^{T}(x)b$$

where *R_i_*(***x***) is the radial basis function (RBFS), and *n_r_* is the number of nodes of the point *x* in the support domain. *p_j_*(***x***) is the polynomial in the space coordinate ***x****^T^* = (*x*, *y*), and *n_p_* represents the number of polynomials. If *n_p_* = 0, it is a single radial basis function (RBFS), otherwise it is an RBF with *n_p_* polynomial basis functions added. Generally, for a one-dimensional problem, the basis function of the polynomial is *p_j_*(*x*) = [1,*x*,...,*x^np^*]*^T^*, and in a two-dimensional problem, the polynomial basis is *p_j_*(*x*) = [1,*x*,*y*,...,*x^np^*,*xy^np^*^−1^,...,*yx^np^*^−1^,*y^np^*]*^T^*. However, using a power function polynomial basis is often inaccurate in solving differential equations. Chebyshev polynomials have important applications in approximation theory. Corresponding interpolation polynomials minimize the Longo phenomenon and provide the best consistent approximation of polynomials in continuous functions. Therefore, this study uses Chebyshev polynomials as interpolation basis functions.

(26) $$P(x)=T(x)={\left\{\begin{array}{ccccc} T_{0}(x) & T_{1}(x) & \cdots & T_{p}(x) & \cdots \end{array}\right\}}^{T}$$

where

(27) $$T_{p}(x)=\cos[p\cos^{-1}(x)]\;,\;p=0,\;1,\;2\cdots v$$

The multi-quadrics (MQ) radial function with shape parameters αc and q are used in this paper.

(28) $$vR_{i}(x)={\left[r_{i}^{2}+{\left(\alpha_{c}d_{c}\right)}^{2}\right]}^{q}$$

where *r_i_* denotes the distance between the supported point *x_J_* (*J* = 1,2,*n_r_*) in the supported domain and calculated node *x_I_*. For the one-dimensional problem in this paper *r_i_* = |*x_J_*-*x_I_*|, *d_c_* is a characteristic length related to the node spacing in the support domain of the compute node. When the nodes are evenly distributed, *d_c_* is the distance between adjacent nodes. Otherwise, dc is the average node spacing within the node distribution domain.

In meshless theory, the size of the local support domain will affect the interpolation accuracy, and a suitable size of the supported domain should be selected [32]. The size of the supported domain of the calculated node can be characterized as follows.

(29) $$vd_{s}=\alpha_{s}d_{c}$$

where *α_s_* represents the scale factor of the support domain.

In order to determine the coefficient vectors ***a*** and ***b*** in Equation (30), a support field for calculated node *x_I_* needs to be formed, which includes *n_r_* field nodes. Let Equation (30) satisfy the calculation of *n* node values around point *x_I_*, which yields *n_r_* linear equations. The matrix of these equations can be expressed as the following form.

(30) $$U_{s}=R_{0}a+T_{n_{t}}b$$

where ***R***_0_ represents the RBFs matrix and ***T****_nt_* is the Chebyshev polynomial matrix [30]. The coefficient vector a of RBFs is expressed as follow.

(31) $$a={\left\{\begin{array}{cccc} a_{1} & a_{2} & \cdots & a_{n_{r}} \end{array}\right\}}^{T}$$

The coefficient vector ***b*** of the Chebyshev polynomial basis function is written as follow:

(32) $$b={\left\{\begin{array}{cccc} b_{1} & b_{2} & \cdots & b_{n_{t}} \end{array}\right\}}^{T}$$

Since there are *n_r_* + *n_t_* unknowns in Equation (35), a unique solution cannot be obtained, so it is necessary to add *n_r_* equations through the following constraints to make the coefficient matrix of the equation system full rank.

(33) $$\sum_{i=1}^{n_{r}}T_{j}(x_{i})a_{i}=T_{n_{t}}^{T}a=0\;,\;j=1,2,\cdots ,n_{t}$$

Combining Equations (35) and (38), the matrix representation of the following system of equations can be generated.

(34) $${\bar{U}}_{s}=\left\{\begin{array}{c} U_{s}\\ 0 \end{array}\right\}=\left[\begin{array}{cc} R_{0} & T_{n_{t}}\\ T_{n_{t}}^{T} & 0 \end{array}\right]\left\{\begin{array}{c} a\\ b \end{array}\right\}=Ga_{0}$$

where

(35) $$a_{0}={\left\{\begin{array}{cccccccc} a_{1} & a_{2} & \cdots & a_{n_{r}} & b_{1} & b_{2} & \cdots & b_{n_{t}} \end{array}\right\}}^{T}$$

(36) $${\bar{U}}_{s}={\left\{\begin{array}{ccccccc} u_{1} & u_{2} & \cdots & u_{n_{r}} & 0 & \cdots & 0 \end{array}\right\}}^{T}$$

From Equation (39)

(37) $$a_{0}=\left\{\begin{array}{c} a\\ b \end{array}\right\}=G^{-1}{\bar{U}}_{s}$$

Substituting Equation (42) into Equation (30)

(38) $$\begin{array}{l} u(x)=R^{T}(x)a+T^{T}(x)b=\left\{\begin{array}{cc} R^{T}(x) & T^{T}(x) \end{array}\right\}\left\{\begin{array}{c} a\\ b \end{array}\right\}\\ \;\;=\left\{\begin{array}{cc} R^{T}(x) & T^{T}(x) \end{array}\right\}G^{-1}{\bar{U}}_{s}={\bar{\Phi }}^{T}(x){\bar{U}}_{s} \end{array}$$

Then, the Chebyshev-RPIM shape function is expressed as follow.

(39) $$\begin{array}{l} {\bar{\Phi }}^{T}(x)=\left\{\begin{array}{cc} R^{T}(x) & T^{T}(x) \end{array}\right\}G^{-1}\\ \;\;\;\;\;=\left\{\begin{array}{ccccccc} \phi_{1}(x) & \phi_{2}(x) & \cdots & \phi_{n_{r}}(x) & \phi_{n_{r}+1}(x) & \cdots & \phi_{n_{r}+n_{t}}(x) \end{array}\right\} \end{array}$$

Delete unnecessary terms in the Chebyshev-RPIM shape function above, and obtain the Chebyshev-RPIM shape function corresponding to the final node displacement.

(40) $$\Phi^{T}(x)=\left\{\begin{array}{cccc} \phi_{1}(x) & \phi_{2}(x) & \cdots & \phi_{n_{r}}(x) \end{array}\right\}$$

Through the above derivation, the displacement components of the nodes can be expressed as follows.

(41) $$u(x)=\Phi^{T}(x)U_{s}=\sum_{i=1}^{n_{r}}\phi_{i}u_{i}$$

(42) $$U_{s}=\{u_{1},u_{2},\cdots ,u_{n_{r}}\}$$

### 2.4. Discretization of Governing Equations and Boundary Conditions

The substructure of the combined structure is discretized using *N* nodes, and the displacement approximation function at node *x_I_* is represented by a Chebyshev-RPIM shape function.

(43) $$U(x_{I})={\left\{\begin{array}{ccccc} u_{I} & v_{I} & w_{I} & \psi_{xI} & \psi_{\theta I} \end{array}\right\}}^{T}=\Phi^{T}(x_{I})U_{s}$$

(44) $$\Phi^{T}(x_{I})=\left[\begin{array}{cccc} \phi_{1}I_{5} & \phi_{2}I_{5} & \cdots & \phi_{Ns}I_{5} \end{array}\right]$$

(45) $$U_{s}={\left[\begin{array}{ccccccccccc} u_{1} & v_{1} & w_{1} & \psi_{x1} & \psi_{\theta 1} & \cdots & u_{Ns} & v_{Ns} & w_{Ns} & \psi_{xNs} & \psi_{\theta Ns} \end{array}\right]}^{T}$$

where *N_s_* represents the number of nodes covered by the support domain, ***I***_5_ represents a 5 × 5 identity matrix.

Substituting Equation (47) into Equation (26) to obtain the discretized governing equation represented by node information.

(46) $$\left(K_{xI}+\omega C_{xI}-\omega^{2}m_{I}\right)U_{s}=0$$

where the nodal matrices ***K****_xI_*, ***C****_xI_* and ***m****_I_* are as follows.

(47) $$K_{xI}=K_{x}\Phi_{I}^{T},\;C_{xI}=C_{x}\Phi_{I}^{T},\;m_{xI}=m_{x}\Phi_{I}^{T}(x=\mathrm{co},\;\mathrm{cy})$$

Similarly, the discrete equations for whole system are obtained by assembling those of each node according to the node number [40].

Substituting Equation (47) into Equation (27) to discretize boundary condition.

(48) $$B_{x}\Phi_{I}^{T}U_{s}=0$$

### 2.5. Continuous Condition

The governing equations and boundary equations of the substructure have been deduced and discretized before, but a complete solution system has not been established. The combined structure can be divided into conical shell and cylindrical shell. According to their geometric characteristics, considering their displacement continuity and physical coordination, the right boundary of the conical shell and the left boundary of the cylindrical shell can be modified as follows.

(49) $$\left\{\begin{array}{c} N_{x}+k_{b}\left(u_{co}-u_{cy}\cos\varphi -w_{cy}\sin\varphi \right)=0\\ N_{x\theta }+k_{b}\left(v_{co}-v_{cy}\right)=0\;\;\;\;\;\;\\ Q_{x}+k_{b}\left(w_{co}+u_{cy}\sin\varphi -w_{cy}\cos\varphi \right)=0\\ M_{x}+k_{b}\left(\psi_{xco}-\psi_{xcy}\right)=0\;\;\;\;\;\;\\ M_{x\theta }+k_{b}\left(\psi_{\theta co}-\psi_{\theta cy}\right)=0\;\;\;\;\;\; \end{array}\right.:\;\mathrm{Right}\;\mathrm{boundary}\;\mathrm{of}\;\mathrm{conical}\;\mathrm{shell}\;\left\{\begin{array}{c} N_{x}-k_{b}\left(u_{cy}-u_{co}\cos\varphi +w_{co}\sin\varphi \right)=0\\ N_{x\theta }-k_{b}\left(v_{cy}-v_{co}\right)=0\;\;\;\;\;\;\\ Q_{x}-k_{b}\left(w_{cy}-u_{co}\sin\varphi -w_{co}\cos\varphi \right)=0\\ M_{x}-k_{b}\left(\psi_{xcy}-\psi_{xco}\right)=0\;\;\;\;\;\;\\ M_{x\theta }-k_{b}\left(\psi_{\theta cy}-\psi_{\theta co}\right)=0\;\;\;\;\;\; \end{array}\right.:\;\mathrm{Left}\;\mathrm{boundary}\;\mathrm{of}\;\mathrm{cylindrical}\;\mathrm{shell}$$

where *k_b_* denotes the connection stiffness between substructures, and symbols *co* and *cy* denote conical and cylindrical shells, respectively. The matrix form of the continuous condition can be written as follows.

(50) $$B_{xco}\Phi_{co}^{T}U_{sco}+K_{12}\Phi_{cy}^{T}U_{scy}=0:\mathrm{Right}\;\mathrm{boundary}\;\mathrm{of}\;\mathrm{conical}\;\mathrm{shell}\;B_{xcy}\Phi_{cy}^{T}U_{scy}+K_{12}\Phi_{co}^{T}U_{sco}=0:\mathrm{Left}\;\mathrm{boundary}\;\mathrm{of}\;\mathrm{cylindrical}\;\mathrm{shell}$$

where ***U****_sco_* and ***U****_scy_* are the displacement vectors of the nodes of the cylindrical shell and the conical shell on the coupling interface, respectively. The coupled stiffness matrices ***K***_12_ and ***K***_21_ are as follows.

(51) $$K_{12}=\left[\begin{array}{ccccc} -k_{b}\cos\varphi & 0 & -k_{b}\sin\varphi & 0 & 0\\ 0 & -k_{b} & 0 & 0 & 0\\ k_{b}\sin\varphi & 0 & -k_{b}\cos\varphi & 0 & 0\\ 0 & 0 & 0 & -k_{b} & 0\\ 0 & 0 & 0 & 0 & -k_{b} \end{array}\right]$$

(52) $$K_{21}=\left[\begin{array}{ccccc} k_{b}\cos\varphi & 0 & -k_{b}\sin\varphi & 0 & 0\\ 0 & k_{b} & 0 & 0 & 0\\ k_{b}\sin\varphi & 0 & k_{b}\cos\varphi & 0 & 0\\ 0 & 0 & 0 & k_{b} & 0\\ 0 & 0 & 0 & 0 & k_{b} \end{array}\right]$$

Finally, matrix assembly is performed to obtain the vibration control equation of the overall structure.

(53) $$(K+\omega C-\omega^{2}m)U=0$$

where

(54) $$K=\left[\begin{array}{cc} K_{coI} & K_{12}\Phi_{co}^{T}\\ K_{21}\Phi_{cy}^{T} & K_{cyI} \end{array}\right]$$

(55) $$C=\left[\begin{array}{cc} C_{coI} & 0\\ 0 & C_{cyI} \end{array}\right]$$

(56) $$m=\left[\begin{array}{cc} m_{coI} & 0\\ 0 & m_{cyI} \end{array}\right]$$

The natural frequency of the conical-cylindrical composite structure in the thermal environment is obtained by the harmonic response method.

## 3. Numerical Results and Discussions

This paper provides a meshless free vibration analysis model of a rotating combined conical-cylindrical shell structure in a thermal environment. The proposed method is compiled with MATLAB software. The number of nodes and the size of the support domain will affect the convergence effect of the algorithm. After obtaining the appropriate support domain size and number of nodes through convergence analysis, the numerical results are compared with finite element software or published literature to ensure the reliability and accuracy of the proposed method. Then, focusing on structural characteristic parameters and the effect of external physics on structural frequencies, some parametric study cases are provided. Unless otherwise stated, the natural frequencies of the considered combined shells are expressed in the dimensionless parameters as $\omega^{*}=\omega R_{1}\sqrt{\rho /E_{2}}$ and the material properties of the layers are given as: *E*_1_ *=* 175 GPa, *E*_2_ *=* 32 GPa, *μ =* 0.25, *G*_12_ *= G*_13_ *=* 12 GPa, *G*_23_ *=* 5.7 GPa, ρ *=* 1760 kg/m^3^, α_11_ *=* 1.2 × 10^−6^, α_22_ *=* 2.3 × 10*^−^*^6^ and α_12_ *=* 0. The symbols C, F, and S are used to represent the tightened boundary conditions, free boundary conditions and simply supported boundary conditions, respectively. The corresponding boundaries are described as follows: C: *k = k_v_ = k_w_ = k_x_ = k_θ_ =* 10^14^. S: *k_u_ = k_v_ = k_w_ = k_θ_ =* 10^14^. *k*_x_ *=* 0, F: *k_u_ = k_v_ = k_w_ = k_x_ = k_θ_ =* 0. Then define boundary rules. For example, CF represents that the boundary of the conical shell segment is a fixed boundary, and the boundary of the cylindrical shell segment is a free boundary.

### 3.1. Verification and Convergence Study

First, according to the basic theory of the meshless method, the key factor affecting the convergence of numerical results is the number of nodes. Therefore, before the numerical comparison and parametric analysis, the advanced convergence analysis is carried out to ensure that the obtained calculation results are stable. Table 1 shows the convergence results of the frequency parameter Ω* (*n* *= m =* 1) of the non-rotating cross-layer cylindrical-conical shell under the classical boundary conditions, and the corresponding geometric dimensions are: *R*_1_ *=* 0.5 m, *L*_1_ *=* 1 m, *L*_2_ *=* 2 m, *h* *=* 0.05 m, *φ* *=* 30°, △*T* *=* 0 K; the lamination scheme is *δ_k_* *=* [0°/90°]. The research results show that, no matter what kind of boundary, when *N* ≥ 9 (*N* is node number), the numerical results are stable and the convergence speed is faster.

**Figure 2:**
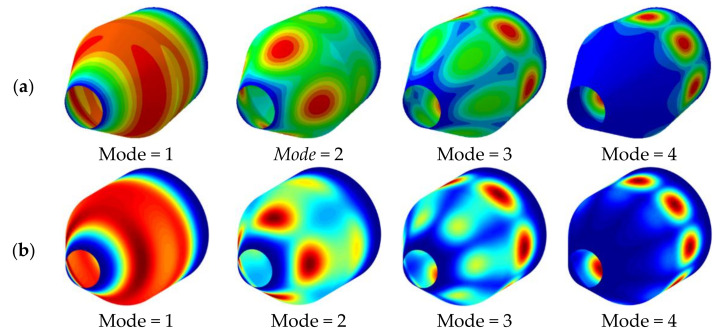
Mode shapes of laminated combined conical-cylindrical shell with CC boundary condition (*m* = 1, *φ* = *π*/6) (**a**) ABAQUS (**b**) Present.

**Figure 3:**
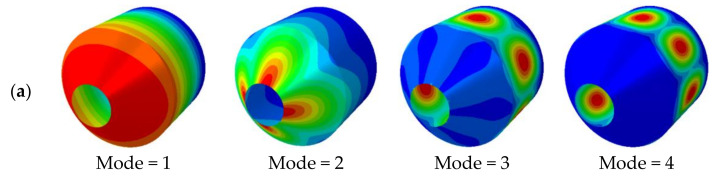

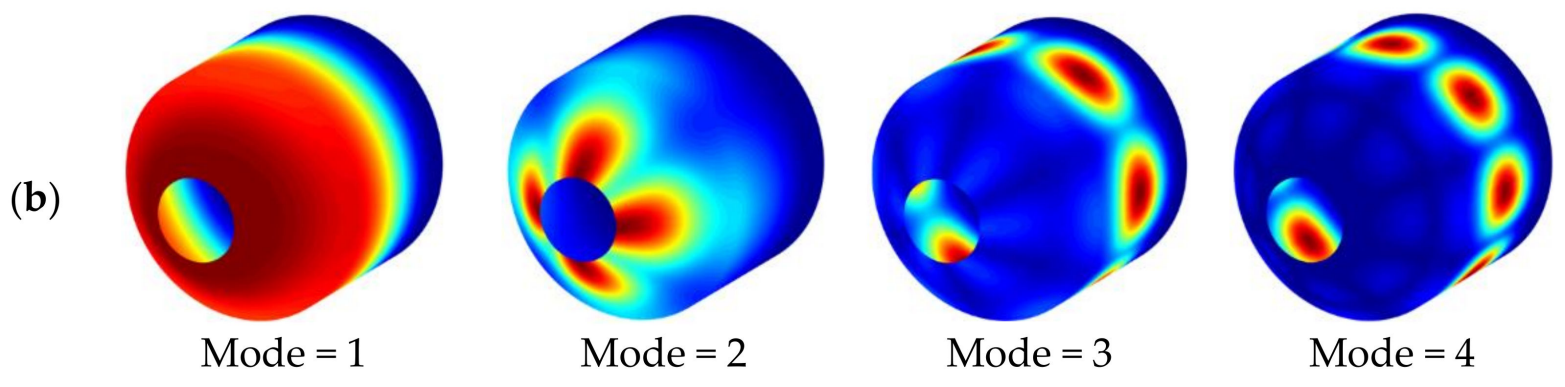
Mode shapes of laminated combined conical-cylindrical shell with FC boundary condition (*m* = 1, *φ* = *π*/4) (**a**) ABAQUS (**b**) Present.

In the previous convergence analysis, it has been determined that the meshless theory is applied to the structural vibration analysis, and the obtained results have good stability. However, it has not been demonstrated whether the obtained results have a high level of confidence. Therefore, it is necessary to further compare the results obtained in this study with the existing publications or the results obtained by the finite element software ABAQUS. Table 2 compares the vibration frequencies of the non-rotating combined conical-cylindrical shell, considering no temperature difference between the inside and outside of the shell. The dimensions of the structure are:*R*_1_ = 0.4226 m, *φ* = π/6, *L*_2_ = *R*_2_ = 1 m and *h* = 0.01 m. The material properties are: *E* = 211 GPa, *ρ* = 7800 kg/m^3^, *μ* = 0.3. The dimensionless frequency of a non-rotating combined conical-cylindrical shell structure is defined as: $\omega^{*}=\omega R_{2}\sqrt{\rho \left(1-\mu^{2}\right)/E}$. The results obtained by the meshless method are compared with the published literature [10] and [15], and the difference between the results obtained by the meshless method and the literature is very small. Table 3 compares the frequency results obtained by different numerical methods for rotating isotropic combined conical-cylindrical shells. The boundary conditions, geometry and Poisson’s ratio of the combined structure are the same as those in Table 2, and the rotational speeds considered are 0.01 rad/s, 100 rad/s, and 500 rad/s, respectively. The comparison results show that the method in this paper is in good agreement with the results in the literature. Finally, it is verified that the model established in this paper can be applied to the structural vibration solution in a thermal environment. In Table 4, the vibration frequency of the non-rotating laminated combined conical-cylindrical shell structure in a thermal environment is analyzed using the finite element software ABAQUS and the method in this paper, respectively. The considered structural geometry is: *R*_1_ = 0.5 m, *R*_2_ = 1.5 m, *L*_2_ = 2 m, *h* = 0.1 m, *N_k_* = [0°/90°/0°]. The temperature change is 50K. The frequencies obtained by these two methods are in good agreement. Figure 2 and Figure 3 represent the mode shapes of laminated combined conical-cylindrical shell, corresponding to the natural frequencies from Table 4. Meanwhile, it is necessary to point out the fact that the following numerical discussion illustrates that this method can be used to analyze structural vibration behavior in thermal environments. All in all, after sufficient comparison, it is proved that the method established in this paper can be applied to the vibration analysis of the rotating composite conical shell and cylindrical shell in a thermal environment.

### 3.2. Numerical Examples

**Figure 4:**
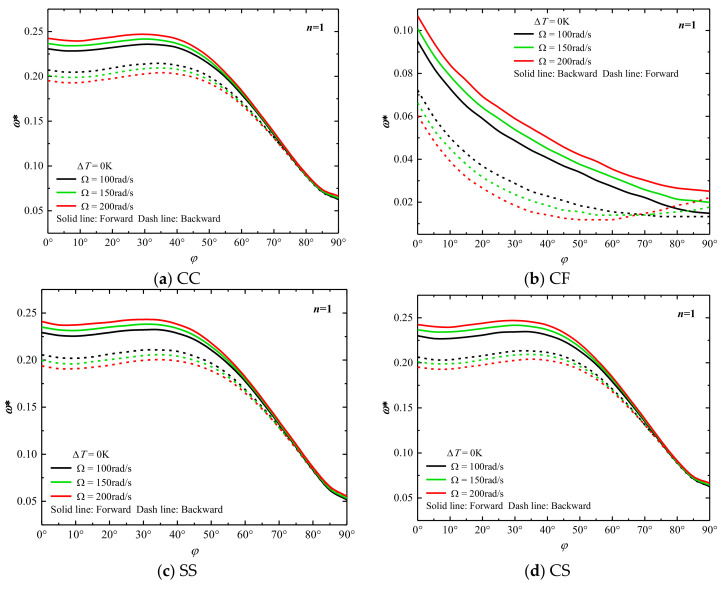
Variation of dimensionless frequencies $\omega^{*}$ of rotating laminated combined conical-cylindrical shell with different semi-vertex angle (*m* = 1).

In Section 3.2, we conduct sufficient comparative verifications to demonstrate that this method can analyze the vibrational behavior of the rotating composite cone-column structure in a thermal environment. First, the effect of semi-vertex of the combined conical-cylindrical shell structure with △*T* *=* 0 K on the natural frequency is studied. The structure shape is: *R*_1_ *=* 0.5 m, *L*_1_ *=* 2 m, *L*_2_ *=* 1 m, *h* *=* 0.05 m, *δ*_k_ *=* [0°/90°/0°/90°]. It can be seen from Figure 4a,c,d that as the half-apex angle of the conical shell increases, the frequency of the combined structure gradually increases slightly first and then decreases significantly under the CC, SS and CS boundary conditions, respectively. At the same time, the difference between the forward wave frequency and the backward wave frequency of the conical-cylindrical composite shell with different rotational speeds is getting smaller and smaller, and the influence of rotational speed is also weakened. As on can see from Figure 4b, under the CF boundary condition, the backward wave frequency of the composite structure corresponding to *Ω* *=* 150 rad/s and *Ω* *=* 200 rad/s will decrease first and then increase with the increase of the half apex angle, and the rest of the natural frequency change curves all decrease. Likewise, with the same rotational speed, the gap between the forward traveling wave and the backward traveling wave of the structure also decreases.

**Figure 5:**
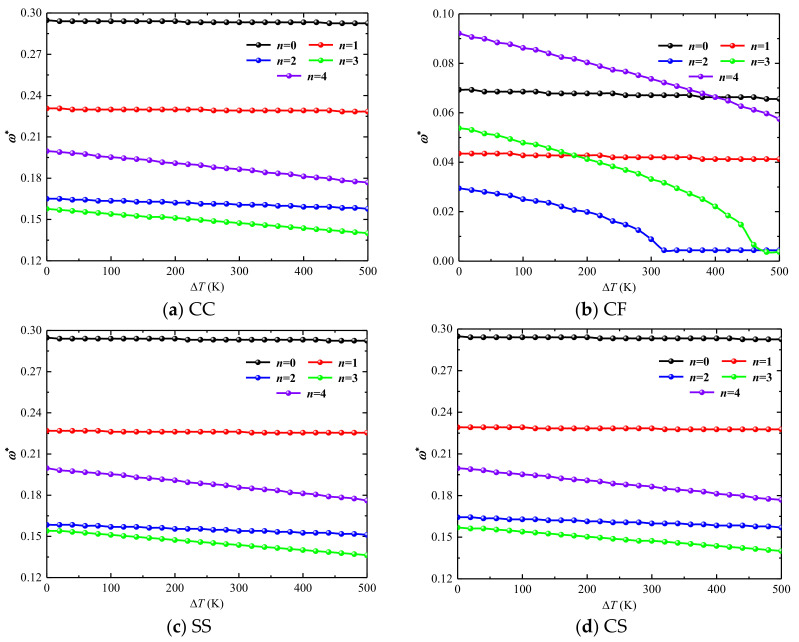
Variation of dimensionless frequencies $\omega^{*}$ of non-rotating laminated combined conical-cylindrical shell subjected to thermal effect (*m* = 1).

Secondly, Figure 5 studies the variation of forward wave frequency with temperature for a combined conical-cylindrical shell with a rotational speed of 50 rad/s. In Figure 5a,c,d we selected the forward wave frequency with the circumferential wave number *n* *=* 1~4 and the axial half-wave number *m* *=* 1 as the research object to discuss its variation with ΔT. The variation interval of the temperature difference is [0 K, 500 K], and the boundary conditions of the studied structures are CC, CF, SS and CS boundary conditions, respectively. The structural geometry parameters are the same as in Figure 4. It is clear from Figure 5b that for the combined structure under the CC, CF, SS and CS boundary conditions, respectively, when *n* *=* 0 and *n* *=* 1, the curve representing the relationship between frequency and temperature difference approaches the horizontal line. At this time, the effect of temperature difference on them is minimal. However, for *n* *=* 2~4, the natural frequency of the structure decreases with increasing temperature difference. Under the CF boundary condition, for *n* *=* 2 and *n* *=* 3 with the increase of the temperature difference, the frequency value first decreases, and then does not change. At this time, at the turning point of the curve, the structure undergoes thermal buckling.

**Figure 6:**
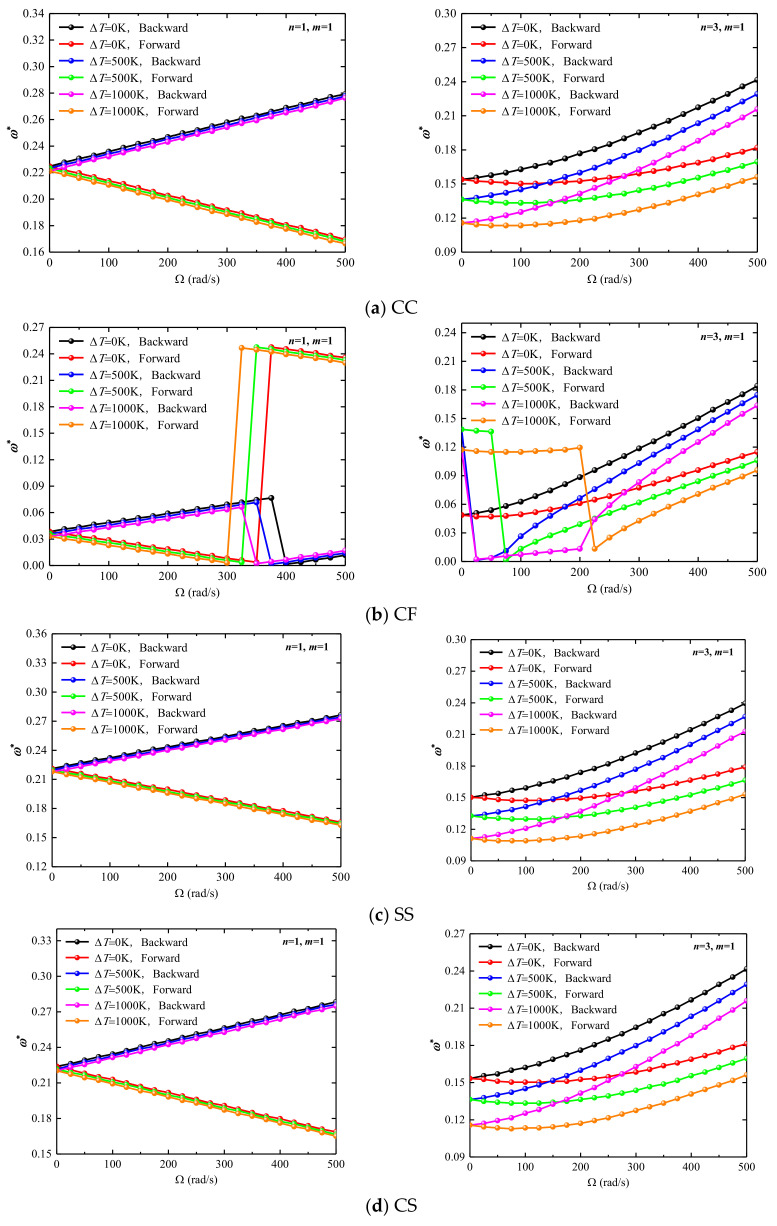
Variation of dimensionless frequencies $\omega^{*}$ of rotating laminated combined conical-cylindrical shell in the thermal environment.

In addition, the effect of the rotational speed on the frequency of the combined structure in the thermal environment is also studied, where three temperature differences are selected as 0 K, 200 K and 1000 K, respectively, the rotational speed variation interval is 0,500], and the boundary condition is CC, CF, SS and CS, respectively. Following the structure of Figure 5 for analysis, the research results are shown in Figure 6. Meanwhile, it is necessary to point out that the $\omega_{f\left(n=1,m=1\right)}^{*}$ and $\omega_{f\left(n=3,m=1\right)}^{*}$ are considered in the following discussion. As can be seen in Figure 6a,c,d, the frequency of all intercepted backward wave frequencies under CC, SS, CS boundary conditions, respectively, increases as the rotational speed increases. However, under CC, CF, SS, CS boundary conditions, respectively, the forward wave frequencies for $\omega_{f\left(n=1,m=1\right)}^{*}$ decrease and for $\omega_{f\left(n=3,m=1\right)}^{*}$ increase with increased rotating speed. As shown in Figure 6b, under CF boundary conditions, the variation tendencies of forward and backward wave frequency are the same as the above other boundary conditions. However, the forward and backward wave frequencies produce model jumping with temperature differences increased, and the above phenomenon can be attributed to thermal buckling.

Finally, Table 5 and Table 6 show the dimensionless frequencies of rotating cross-ply combined conical-cylindrical shell with various geometry and boundary condition in thermal environment. The geometrical parameters of the structure and the temperature difference are given in the table header, and the lamination scheme is [0°/90°/0°]. It can be seen from Table 5 that as the length of the cylindrical shell increases, the stiffness of the structure decreases, and both the forward wave frequency and the backward wave frequency gradually decrease. The rules in Table 6 are the same as those in Figure 4, and thus are not repeated here. These results are valuable to designers and serve as benchmarks for future numerical studies.

## 4. Conclusions

This paper focuses on the free vibration analysis of laminated combined conical-cylindrical shell rotating in a thermal environment. The equations of motion of the whole system are derived by combining the equations of individual substructures obtained using Hamilton’s principle, in which the effects of temperature change, centrifugal and Coriolis forces are taken into account. For numerical calculation of equations of motions, the meshfree strong form method using TRPIM shape function is applied. Through the convergence study, the number of node is determined. The accuracy and reliability of the proposed method are verified through comparison with the results of finite element program. Finally, the effects of parameters such as geometric dimensions, rotating speed and temperature change on the free vibration of combined conical-cylindrical shell are investigated through some numerical examples. The conclusions obtained in this study are as follows:

(1) The meshless Chebyshev-PRIM technique is effective and has relatively high accuracy in the vibration solution of rotating structures. This method has the advantage of fast convergence, and relatively accurate results can be obtained with a smaller number of nodes.
(2) The increase of the half-apex angle of the conical shell reduces the structural rigidity, so the structural frequency decreases. For the combined structure under the CC boundary, after the cone angle increases to a certain extent, the effect of the rotational speed will decrease, and the frequencies corresponding to different rotational speeds will gradually approach.
(3) If the temperature is too high, thermal stress is accumulated inside the structure, the stiffness of the structure is reduced, and the frequency of the combined structure will also decrease. For the boundary conditions with weakened constraints, such as the CF boundary, thermal buckling also occurs with the increase of the temperature difference.

## Figures and Tables

**Table 1:** Variation of dimensionless frequencies on number of nodes (*m* = 1).

*N*	CC		SS		FC		CF	
	*n* = 1	*n* = 2	*n* = 1	*n* = 2	*n* = 1	*n* = 2	*n* = 1	*n* = 2
5	0.2328	0.1812	0.2232	0.1754	0.1171	0.0803	0.0494	0.0339
6	0.2284	0.1790	0.2262	0.1761	0.1127	0.0825	0.0464	0.0317
7	0.2306	0.1805	0.2255	0.1754	0.1142	0.0833	0.0457	0.0317
8	0.2299	0.1798	0.2262	0.1754	0.1135	0.0840	0.0464	0.0324
9	0.2299	0.1798	0.2262	0.1754	0.1135	0.0840	0.0457	0.0317
10	0.2299	0.1798	0.2262	0.1754	0.1135	0.0840	0.0457	0.0317
11	0.2299	0.1798	0.2262	0.1754	0.1135	0.0840	0.0457	0.0317
12	0.2299	0.1798	0.2262	0.1754	0.1135	0.0840	0.0457	0.0317
13	0.2299	0.1798	0.2262	0.1754	0.1135	0.0840	0.0457	0.0317
14	0.2299	0.1798	0.2262	0.1754	0.1135	0.0840	0.0457	0.0317
15	0.2299	0.1798	0.2262	0.1754	0.1135	0.0840	0.0457	0.0317
16	0.2299	0.1798	0.2262	0.1754	0.1135	0.0840	0.0457	0.0317
17	0.2299	0.1798	0.2262	0.1754	0.1135	0.0840	0.0457	0.0317
18	0.2299	0.1798	0.2262	0.1754	0.1135	0.0840	0.0457	0.0317
19	0.2299	0.1798	0.2262	0.1754	0.1135	0.0840	0.0457	0.0317
20	0.2299	0.1798	0.2262	0.1754	0.1135	0.0840	0.0457	0.0317
21	0.2299	0.1798	0.2262	0.1754	0.1135	0.0840	0.0457	0.0317

**Table 2:** Comparison of dimensionless frequencies for non-rotating isotropic combined conical-cylindrical shell with F-C boundary condition (*μ* = 0.3).

*m*	*n* = 0		*n* = 1		*n* = 2	
	FEM	Present	FEM	Present	FEM	Present
1	0.50375	0.50305	0.29287	0.29279	0.10203	0.09996
2	0.60986	0.60985	0.63581	0.63506	0.50290	0.50217
3	0.93092	0.93082	0.81123	0.81141	0.69148	0.69116
4	0.95632	0.95612	0.93088	0.93137	0.85890	0.85888
5	0.97160	0.97134	0.94850	0.95183	0.91607	0.91544
6	1.01188	1.01142	0.99145	0.99156	0.96048	0.96007
	***n* = 3**		***n* = 4**		***n* = 5**	
	**FEM**	**Present**	**FEM**	**Present**	**FEM**	**Present**
1	0.09377	0.08750	0.14460	0.14441	0.20390	0.19930
2	0.39220	0.39115	0.33034	0.32996	0.29633	0.29579
3	0.51518	0.51434	0.39562	0.39537	0.37623	0.37013
4	0.75359	0.75289	0.64458	0.64594	0.58167	0.57874
5	0.79698	0.79629	0.69114	0.69248	0.61422	0.61285
6	0.91939	0.91893	0.87194	0.87098	0.81980	0.81642

**Table 3:** Comparison of dimensionless frequencies for a rotating isotropic combined conical-cylindrical shell.

Ω*	*n*	FEM		Present	
		w*_b_	w*_f_	w*_b_	w*_f_
0.01 rad/s	1	0.5264	0.5264	0.5267	0.5267
	2	0.3769	0.3769	0.3774	0.3774
	3	0.2873	0.2873	0.2869	0.2869
	4	0.236	0.236	0.2363	0.2363
	5	0.2231	0.2231	0.2246	0.2246
	6	0.2474	0.2474	0.2469	0.2469
100 rad/s	1	0.5430	0.5097	0.5432	0.5103
	2	0.3906	0.3648	0.3904	0.3645
	3	0.3005	0.2816	0.3010	0.2822
	4	0.2527	0.2383	0.2528	0.2387
	5	0.2455	0.234	0.2469	0.2352
	6	0.2747	0.2647	0.2740	0.2645
500 rad/s	1	0.6085	0.4422	0.6090	0.4421
	2	0.4605	0.3308	0.4609	0.3304
	3	0.4174	0.322	0.4174	0.3222
	4	0.4484	0.3756	0.4480	0.3762
	5	0.5212	0.4629	0.5220	0.4633
	6	0.6157	0.5612	0.6161	0.5608

**Table 4:** Comparison of natural frequencies for non-rotating isotropic combined conical-cylindrical shell in thermal environment (Δ*T* = 50 K).

*φ*	Mode	CC			CS			FC		
		FEM	Present	Diff,%	FEM	Present	Diff,%	FEM	Present	Diff,%
π/6	1	234.96	237.01	0.872	227.4	228.08	0.299	120.2	119.54	−0.549
	2	250.73	251.66	0.371	227.84	228.85	0.443	133.66	133.66	0
	3	252.19	254.21	0.801	244.34	243.77	−0.233	234.47	234.16	−0.132
	4	265.13	264.69	−0.166	247.19	247.93	0.299	240.92	241.13	0.087
	5	272.86	274.39	0.561	265.13	264.69	−0.166	270.66	270.72	0.022
	6	285.89	286.71	0.287	281.3	282.09	0.281	272.89	274.28	0.509
π/4	1	270.23	272.39	0.799	239.64	239.73	0.038	145.93	145.81	−0.082
	2	281.53	283.4	0.664	250.99	250.54	−0.179	152.71	152.7	−0.007
	3	293.78	293.32	−0.157	271.81	272.52	0.261	256.96	256.67	−0.113
	4	294.13	296.48	0.799	293.78	293.32	−0.157	268.27	270.41	0.798
	5	319.31	321.55	0.702	302.2	300.76	−0.477	281.31	283.08	0.629
	6	328.18	329.1	0.28	312.49	314.21	0.55	305.66	306.68	0.334

**Table 5:** Dimensionless frequencies of rotating laminated combined conical-cylindrical shell with various length ratio in thermal environment. (*L*_1_ = 1, *R*_1_ = 0.5 m, *h* = 0.05 m, *m* = 1, *φ* = 30˚, Δ*T* = 50 K).

*L*_2_/*L*_1_	Ω, rad/s	*n*	Forward				Backward			
			CC	SS	CF	FC	CC	SS	CF	FC
0.5	50	1	0.4723	0.4561	0.1127	0.2549	0.4826	0.4664	0.1230	0.2667
		2	0.3809	0.3478	0.0847	0.1157	0.3883	0.3559	0.0928	0.1245
		3	0.3544	0.3153	0.1341	0.1709	0.3595	0.3212	0.1400	0.1776
	100	1	0.4671	0.4509	0.1083	0.2490	0.4877	0.4715	0.1282	0.2726
		2	0.3772	0.3448	0.0818	0.1120	0.3927	0.3595	0.0980	0.1297
		3	0.3522	0.3139	0.1326	0.1702	0.3640	0.3249	0.1459	0.1820
1	50	1	0.3735	0.3618	0.0759	0.1835	0.3846	0.3728	0.0862	0.1945
		2	0.3448	0.3161	0.0361	0.0921	0.3536	0.3249	0.0449	0.1009
		3	0.3404	0.3028	0.0663	0.1687	0.3470	0.3087	0.0729	0.1754
	100	1	0.3676	0.3559	0.0707	0.1776	0.3905	0.3780	0.0914	0.2004
		2	0.3411	0.3124	0.0332	0.0884	0.3581	0.3293	0.0508	0.1061
		3	0.3382	0.3006	0.0670	0.1680	0.3507	0.3131	0.0803	0.1805
1.5	50	1	0.2896	0.2829	0.0553	0.1392	0.3006	0.2947	0.0663	0.1510
		2	0.2505	0.2358	0.0258	0.0781	0.2593	0.2446	0.0346	0.0869
		3	0.2218	0.2019	0.0597	0.1665	0.2291	0.2092	0.0670	0.1724
	100	1	0.2837	0.2778	0.0501	0.1334	0.3065	0.2999	0.0715	0.1562
		2	0.2461	0.2313	0.0236	0.0744	0.2645	0.2498	0.0420	0.0928
		3	0.2203	0.2004	0.0612	0.1650	0.2336	0.2137	0.0752	0.1776
2	50	1	0.2328	0.2284	0.0420	0.1105	0.2439	0.2402	0.0530	0.1223
		2	0.1864	0.1783	0.0214	0.0700	0.1952	0.1871	0.0302	0.0788
		3	0.1606	0.1496	0.0575	0.1540	0.1672	0.1569	0.0641	0.1606
	100	1	0.2269	0.2225	0.0368	0.1046	0.2498	0.2453	0.0589	0.1282
		2	0.1820	0.1739	0.0192	0.0670	0.2004	0.1923	0.0376	0.0847
		3	0.1584	0.1481	0.0589	0.1525	0.1724	0.1621	0.0729	0.1658

**Table 6:** Dimensionless frequencies of rotating laminated combined conical-cylindrical shell with various semi-vertex angle in thermal environment. (*L*_1_ = 0.5 m, *L*_2_ = 2 m, *R*_1_ = 0.5 m, *h* = 0.1 m, *m* = 1, Δ*T* = 50 K).

*φ*	Ω, rad/s	*n*	Forward				Backward			
			CC	SS	CF	FC	CC	SS	CF	FC
π/6	50	1	0.2763	0.2645	0.0729	0.1260	0.2881	0.2763	0.0840	0.1378
		2	0.2269	0.2085	0.0766	0.1444	0.2365	0.2173	0.0855	0.1532
		3	0.2807	0.2652	0.2033	0.2763	0.2881	0.2726	0.2107	0.2829
	100	1	0.2704	0.2593	0.0670	0.1201	0.2940	0.2822	0.0899	0.1437
		2	0.2225	0.2041	0.0729	0.1400	0.2417	0.2225	0.0914	0.1584
		3	0.2785	0.2630	0.2019	0.2741	0.2925	0.2770	0.2159	0.2873
π/4	50	1	0.2859	0.2756	0.0589	0.1297	0.2969	0.2866	0.0700	0.1415
		2	0.2306	0.2115	0.0597	0.1606	0.2402	0.2210	0.0685	0.1695
		3	0.2549	0.2365	0.1599	0.2520	0.2616	0.2431	0.1665	0.2586
	100	1	0.2800	0.2697	0.0538	0.1238	0.3028	0.2925	0.0752	0.1474
		2	0.2269	0.2078	0.0560	0.1562	0.2453	0.2255	0.0744	0.1746
		3	0.2527	0.2343	0.1577	0.2498	0.2667	0.2476	0.1717	0.2638
π/3	50	1	0.2918	0.2822	0.0494	0.1319	0.3028	0.2940	0.0597	0.1437
		2	0.2343	0.2144	0.0501	0.1857	0.2431	0.2232	0.0597	0.1945
		3	0.2424	0.2218	0.1356	0.2394	0.2498	0.2291	0.1422	0.2468
	100	1	0.2859	0.2770	0.0442	0.1260	0.3087	0.2999	0.0648	0.1496
		2	0.2299	0.2100	0.0472	0.1812	0.2483	0.2284	0.0656	0.1989
		3	0.2402	0.2196	0.1341	0.2372	0.2542	0.2336	0.1481	0.2512
π/2	50	1	0.2962	0.2873	0.0427	0.1334	0.3080	0.2991	0.0516	0.1451
		2	0.2380	0.2181	0.0457	0.2092	0.2476	0.2269	0.0545	0.2181
		3	0.2372	0.2144	0.1194	0.2321	0.2439	0.2218	0.1267	0.2387
	100	1	0.2903	0.2814	0.0383	0.1275	0.3139	0.3043	0.0560	0.1510
		2	0.2343	0.2137	0.0427	0.2048	0.2520	0.2321	0.0604	0.2232
		3	0.2350	0.2129	0.1186	0.2299	0.2483	0.2262	0.1326	0.2439
